# Catheter-Based Intramyocardial Delivery (NavX) of Adenovirus Achieves Safe and Accurate Gene Transfer in Pigs

**DOI:** 10.1371/journal.pone.0053007

**Published:** 2013-01-03

**Authors:** Bo Chen, Zhengxian Tao, Yingming Zhao, Hongwu Chen, Yonghong Yong, Xiang Liu, Hua Wang, Zuze Wu, Zhijian Yang, Li Yuan

**Affiliations:** 1 Department of Cardiology, the First Affiliated Hospital of Nanjing Medical University, Nanjing, Jiangsu, The People’s Republic of China; 2 Department of Technological Development, MicroPort Medical (Shanghai) Co. Ltd., Shanghai, The People’s Republic of China; 3 Department of Experimental Hematology, Beijing Institute of Radiation Medicine of China, Academy of Military Medical Sciences, Beijing, The People’s Republic of China; 4 Department of Biochemistry and Molecular Biology, Nanjing Medical University, Nanjing, Jiangsu, The People’s Republic of China; University of Nevada School of Medicine, United States of America

## Abstract

**Background:**

Hepatocyte growth factor (HGF) is one of the major angiogenic factors being studied for the treatment of ischemic heart diseases. Our previous study demonstrated adenovirus-HGF was effective in myocardial ischemia models. The first clinical safety study showed a positive effect in patients with severe and diffused triple coronary disease.

**Methods:**

12 Pigs were randomized (1∶1) to receive HGF, which was administered as five injections into the infarcted myocardium, or saline (control group). The injections were guided by EnSite NavX left ventricular electroanatomical mapping.

**Results:**

The catheter-based injections caused no pericardial effusion, malignant arrhythmia or death. During mapping and injection, alanine aminotransferase, aspartate aminotransferase, blood urea nitrogen, serum creatinine and creatine kinase-MB levels have no significant increase as compared to those before and after the injection in HGF group(P>0.05). HGF group has high HGF expression with Western blot, less myocardial infarct sizes by electroanatomical mapping (HGF group versus after saline group, 5.28±0.55 cm^2^ versus 9.06±1.06 cm^2^, P<0.01), better cardiac function with Gated-Single Photon Emission Computed Tomography compared with those in saline group. Histological, strongly increased lectin–positive microvessels and microvessel density were found in the myocardial ischemic regions in HGF group.

**Conclusion:**

Intramyocardial injection guided by NavX system provides a method of feasible and safe percutaneous gene transfer to myocardial infarct regions.

## Introduction

Therapeutic angiogenesis induced by vascular growth factors may represent a novel approach to the treatment of ischemic heart disease. Because of its potential angiogenic, antiapoptotic, antifibrotic and stem cell-recruiting effects, HGF has been a subject drawing more and more attention in field of cardiovascular diseases [1]. We previously constructed a replication-deficient adenovirus carrying the HGF gene (Ad-HGF) [2]. In experimental animal models, this adenovirus mediates high levels of expression of human HGF, subsequently leading to formation of neovasculature and improvement of post-infarct heart function [2], [3]. Preclinical research demonstrated that Ad-HGF was effective in both acute and chronic myocardial ischemic models and no toxicity or mutations have been observed in rat, pig or rhesus monkey [4], [5], [6]. Our phase I clinical study showed that it was safe and effective using an adenoviral gene transfer vector to deliver the human HGF to individuals with clinically significant coronary artery disease [7], [8]. Ad-HGF has been delivered either by intracoronary infusion or by direct intramyocardial injection following open chest thoracotomy [3], [9]. However, the most safe and feasible route of delivery of angiogenic factors into the myocardium remains unknown. Ideally, a catheter-based intramyocardial injection system could provide accurate intramyocardial injection of angiogenic factors (genes or peptides) without the need for surgery and general anesthesia. We developed a set of percutaneous intramyocardial injection system guided by EnSite NavX (NavX) for the gene and cell therapies in patients with coronary ischemia.

The present study describes a new electromechanical-based platform system for intramyocardial injection that may be used to achieve therapeutic angiogenesis and improvement of cardiac function. Our system includes an injecting catheter that has a 27 gauge needle for intramyocardial delivery of potentially therapeutic agents, and its intellectual property is protected in China (patent number: CN 101536902A).

The purpose of this study was to evaluate the feasibility and safety of electromechanical guidance for the catheter-based intramyocardial injection system in ischemic myocardium, and assess the accuracy of the system to locate, in real time, the sites of intramyocardial injection. Furthermore, gene transferring (GTx) into ischemic myocardium can lead to successful gene expression at high levels and improvement of cardiac function, as proven by this integrated catheter system.

## Materials and Methods

### Injection System

**Figure 1:**
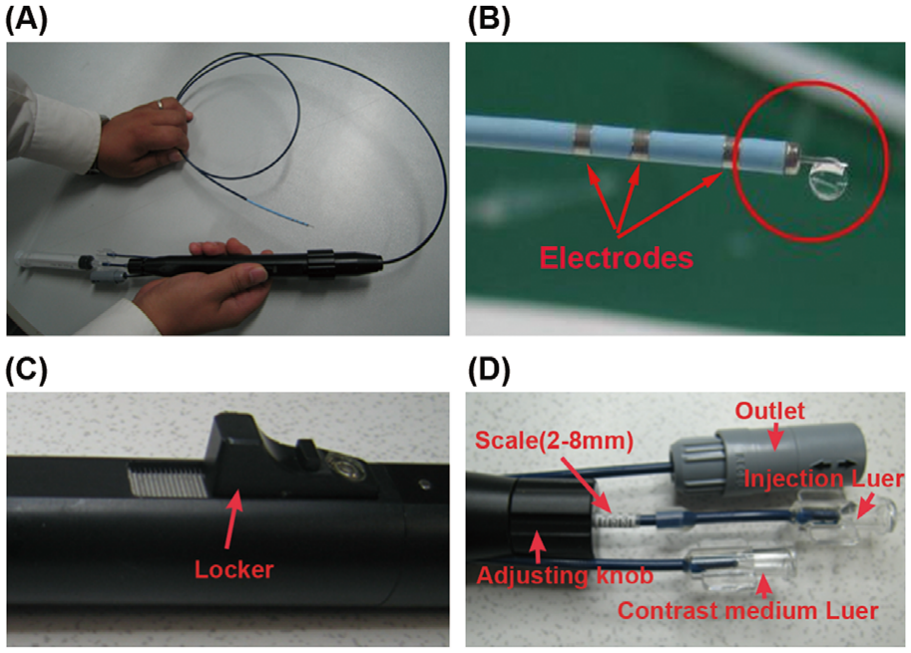
The intramyocardial injection system. (A)The injection device comprises a catheter and handle. The catheter involves in mapping and injection functions. (B) The distal end of the catheter is equipped with tip and ring electrodes, a location sensor and a 27 guage needle designed for transendocardial injection. The red circle marks out a drop of saline injected through the needle tip. (C) The locker fixed the needle whose length was selected. (D) The adjusting knob controls the length of needle into myocardium. Two Luer fit for connection to an auto-syringe pump and a syringe of contrast medium, respectively.

Intramyocardial injection system includes a catheter and a handle (Fig. 1A). The catheter consists of a 27 gauge needle. Importantly, the distal tip of injection catheter is fixed with a headend and a three-loop electrode to detect local signals. Once ventricular reconstruction is completed, endocardial injection could be accomplished by the injection catheter. The handle is equipped with an auto-syringe pump, adjusting knob and a locker. The pump could control injection doses (0.05–0.3 ml each time, difference≤1%), depth (3–10 mm) and speed (2–25 seconds) (Figure 1B). The needle “dead space” is approximately 0.99 cc. The needle depth into myocardium is controlled by the adjusting knob and locker. The injection handle has an injection Luer fitting for connection to a 1–4 cc syringe. Standard operation of the syringe attached to the injection handle delivers the fluid into the myocardium. The exact catheter tip location, orientation, and the injection sites are indicated in real time on the ventricular mapping, and local signals are traced to ensure catheter stability and optimal endocardial contact.

To determine the injection speed in various doses, methylene blue dye (MB) was injected into porcine normal heart ex vivo. 3 mm, 5 mm, 10 mm into myocardium was designed for the injection depth. 0.2 ml and 0.3 ml MB was injected into myocardium at the speed of 2 s, 5 s, 10 s, 15 s, 20 s, and 25 s, respectively. A total of 180 injections were conducted in 14 porcine hearts. The MB overflow and bubbling from the site of endocardial injection were checked after the injection.

### Construction of Adenoviral Vector

Adenoviral vectors constructed for previous experiments were used in this study [2]. HGF cDNA was amplified by polymerase chain reaction using human placenta cDNA library as template. Replication-deficient (E1, E3 deleted) adenoviral vectors containing the human hepatocyte growth factor (Ad-HGF) was constructed on basis of the pAdEasy-1 system (Stratagene, La Jolla, CA) following the manufacturer’s instructions. The recombinant replication defective adenovirus was purified by cesium chloride gradient ultracentrifugation. The final plaque-forming units (pfu) were determined by titration on HEK293 cells under an agarose overlay.

### Animal Preparation

Eighteen mini-pigs (male, 3–4 months old, 36±3.4 kg body weight) were housed in the animal care facility of Nanjing Medical University. All animals received humane care in compliance with the Guideline for Care and Use of Laboratory Animals published by Jiangsu Province, P. R. China. The approval was granted by Nanjing Medical University ethical review board. The myocardial infarction models constructed for previous experiments were used in this study [10]. Three pigs died during the ligation of LAD. Twelve pigs were randomly divided into two groups of 6 each: Ad-HGF group (HGF group), and HEPES saline injected group (saline group). The other three animals were used to assess injection accuracy.

### Left Ventricular Mapping and Injection Accuracy

The guiding NavX system utilizes a 5.68 kHz constant low-current locator signal and sensor-tipped catheters to locate the catheter position in space [11], [12]. Three-dimensional (3D) electromechanical maps of the left ventricle (LV) can be used to help differentiate between healthy and infarcted myocardium and identify most ischemic target regions without using fluoroscopy [13], [14]. Technical details of three-dimensional mapping system integrated as a part of the EnSite system have been described previously [11]. Briefly, to obtain three-dimensional left ventricular geometry, a roving mapping catheter is swept throughout the chamber, defining endocardial boundaries. Two locatable catheters (reference and mapping catheters) were introduced into the heart under fluoroscopic guidance. The location of the tip of the roving mapping catheter in left ventricular chamber was recorded according to the fixed reference catheter positioned at the coronary sinus. After the left ventricular geometry was created under the guidence of EnSite NavX (St. Jude Medicine, Inc., USA), electrograms were recorded by sampling the location of a roving catheter throughout the left ventricular endocardium. The average voltages were analyzed by diagnostic landmarking tools. Colors represent local voltage. Gray and purple respectively indicated low (≤0.5 mV) and normal voltage (≥2.0 mV). The region between gray and purple was peri-infarct zone. To analyze the voltage of scar and peri-infarct zone,over 70 points was mapped by two skilled operators in the region between gray and purple. The low voltage area was measured by diagnostic landmarking tools for detecting infarct sizes.

To estimate whether the sites of intramyocardial injection was accurately located in real time, a total of 1 ml MB was injected at the volume of 0.2 ml and speed of 20 s each time into five sites of the myocardium around the peri-infarct zone of three porcine infarcted hearts in vivo guided by NavX.

### NavX Gene Transfer

Four weeks after the ligation of the LAD, the animals were randomly divided into two groups (n = 6): Ad-HGF injected group (HGF group), and HEPES saline injected group (saline group). The saline animals were used as the negative controls. An intramyocardial injection system was used to percutaneous endocardial injection of adenoviral vector. After ≥70 points had been mapped by NavX, the injection catheter was navigated into the infarcted region, and five different sites of intramyocardial injections were determined around the infarct zone (3 mm depth, 20 s, 0.2 ml each, 5 seconds interval, total volume 1.0 ml, ≥5 mm apart from each other). A total of 1×10^10^ pfu of viral titers in 1.0 ml of HEPES saline (pH 7.4) were used. The quantity of Ad-HGF was selected by previous studies [6]. Identical doses of saline were injected into control animals. Four weeks after gene transfer (GTx), the size of scar and average voltage were analyzed through the above method. During mapping and injection, the time and doses of radiation were measured, and then the porcine hearts were collected.

### Clinical Chemistry Analysis

Safety was evaluated by animal survival, pericardial effusion, heart rhythm, blood tests. Survival were continuously monitored and recorded during the 8 weeks study period. Heart rhythm was monitored with the surface electrocardiogram and intracardiac electrical record during mapping and injection. Blood samples were collected at selected points of time. The white blood cell (WBC), alanine aminotransferase (ALT), aspartate aminotransferase (AST), blood urea nitrogen (Bun), creatinine (Cr) and creatine kinase-MB (CK-MB) in serum were measured at the Central Laboratory in the First Affiliated Hospital of Nanjing Medical University.

### SPECT Analysis

Gated-single photon emission computed tomography (SPECT) was performed in the pigs with a commercially available system (ECAM+; Siemens, Germany). Left ventricular end-diastolic volume (LVEDV), left ventricular end-systolic volume (LVESV) and left ventricular ejection fraction (LVEF) were determined by QGS software. SPECT analysis has been described previously [10]. In brief, the myocardial perfusion score was calculated as follows: The left ventricular cavity was divided into 17 segments for assessment of the myocardium [15]. Seven segments were assigned to the LAD distribution area. The five-point scoring used to grade each segment was as follows: 0 = absent perfusion; 1 = severe hypoperfusion; 2 = moderate hypoperfusion; 3 = mild hypoperfusion; and 4 = normal perfusion. The normal total scores of LAD areas were 28 [16].

### Western Blotting Analysis

Technical details of Western blotting analysis have been described previously [10]. HGF, β-actin and the secondary goat anti-mouse IgG-AP antibody (Santa Cruz Biotechnology, USA) were used in this study.

### Histological and Immunohistochemical Analysis

Transverse sections of hearts were obtained at intervals of 5 mm in the upward direction from the heart apex. Then sections were fixed in 10% formalin, embedded in paraffin and sectioned. Serial sections were made from the apex of the heart to the site of the ligation. Sections were stained with specific antibodies against lectin, Ki67 (Santa Cruz Biotechnology) antibodies. Nuclei were stained with 4′,6′-diamino-2-phenilindole (DAPI). Lectin-FITC (Santa Cruz Biotechnology) was used to visualize the blood vessels. The immunohistochemical staining was carried out according to the manufacturer’s instructions, and signals were visualized by incubating the sections with Alexa Fluor-488 or −555-labeled secondary antibody (Molecular Probes Inc., Eugene, OR, USA).

For quantification of the vessel-densities in the myocardium, four sections were randomly selected from each group, and six visual fields from each section were observed. As a surrogate for vessel counting, lectin- and Ki67-positive vessel density was determined by optical density measurements using the QWIN100 immunohistochemical imaging analyzer system (Leica Co., Germany).

### Statistical Analyses

Statistical analysis was performed with SPSS 10.0 software. Student’s t-tests and one-way ANOVA testing were used to compare the differences among groups, with statistical significance considered if P<0.05. The data are presented as mean ± standard deviation (SD).

## Results

### Injection Speed and Depth

To determine correlation between doses and the depth and speed of injection via our catheter-based injector, MB was injected into fourteen porcine normal hearts in vitro. When 0.2 ml MB was injected into porcine myocardium at the speed of 2 s and 5 s, overflow and bubbling were significantly observed in the surface of endocardium; while at the speed of 10 s and 15 s, overflow was not significantly observed, and no bubbling occured; at 20 s and 25 s, there observed neither overflow nor bubbling in the sites of injection. While injecting 0.3 ml MB, the MB significantly flowed outward the endocardium at the speed of 2 s, 5 s, and 10 s; less MB was observed at the speed of 15 s and 20 s; the overflow was not detected in the endocardium at the speed of 25 s. Once injection time took longer than 15 s, the bubbling did not occur in the surface of endocardium. No MB staining was observed in the pericardium injected at depths of 3 mm and 5 mm. For depth of 10 mm, MB staining occurred in the pericardium of five pigs (0.2 ml MB: two pigs, 0.3 ml MB: three pigs). Overall, when injection of 0.2 ml and 0.3 ml doses took 20 s, the overflow and bubbling of MB could not be detected in the sites of injected endocardium; injection into myocardium at depths of 3 mm and 5 mm could not cause pericardium perforation (Table 1). Therefore, injection into myocardium at the speed of 20 s with the depth of 3 mm was selected in the subsequent GTx guided by NavX *in vivo*.

**Table 1:** Injection of speed and depth.

Time/Volume/Depth	Overflow	Bubbling	MB staining
2 s or 5 s/0.2 or 0.3 ml/3 or 5 mm	Yes	Yes	No
10 or 15 s/0.2 or 0.3 ml/3 or 5 mm	Yes	No	No
20 or 25 s/0.2 or 0.3 ml/3 or 5 mm	No	No	No
2 or 5 or 10 or 15 or 20 or 25 s/0.2 or 0.3 ml/10 mm	No	No	Yes

Yes: the overflow and bubbling were detected in the endocardium. No: the overflow and bubbling were not detected in the endocardium, or MB staining not in the pericardium.

### Injection Accuracy

**Figure 2:**
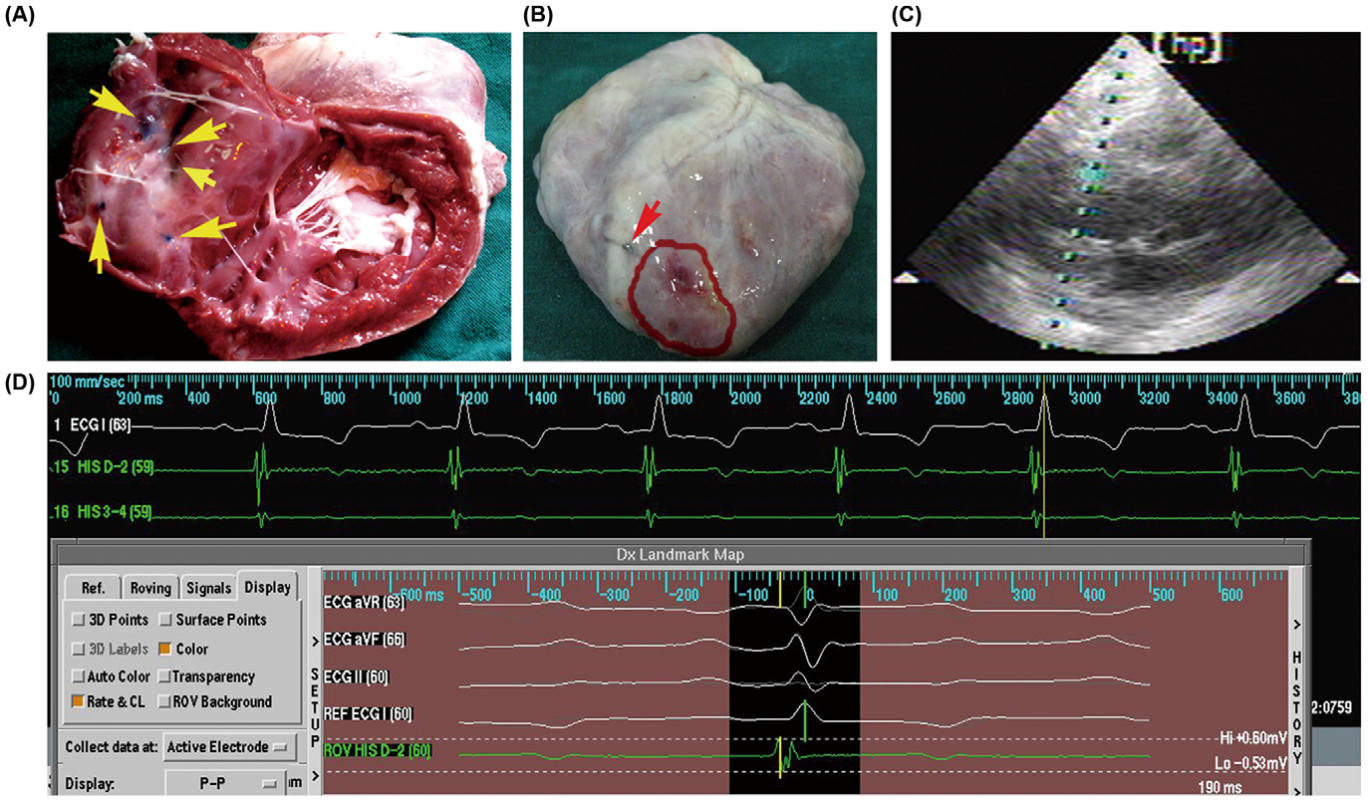
The accuracy and safety of injection. (A), (B) Representative images of methylene-blue injection. The yellow arrows pointed to injection sites, all of the sites were around the desired myocardial ischemic region (pale region). The red arrow pointed to the ligation site of the LAD. The myocardial ischemic region was marked with red curve. No MB staining was detected in the pericardium. (C) Representative image of the echocardiography. During the mapping and injection, the pericardial effusion was not detected in two groups. (D) Representative image of the surface electrocardiogram and intracardiac electrical recording. The operation did not cause malignant arrhythmia in real time.

To estimate the accuracy of the intramyocardial injection system, MB was injected at the volume of 0.2 ml each time into other three porcine infarcted hearts in vivo under the guidance of NavX. The five injection points were all around myocardial infarction areas (Fig. 2A), and the MB staining was not detected in the pericardium (Fig. 2B).

### Injection Safety

WBC, ALT, AST, Bun and Cr levels were not significantly changed after the injection compared to those before the injection in both groups (P>0.05, Table 2). CK-MB was significantly increased after 6, 24 hours and 3 days of LAD ligation compared to that before ligation (P<0.05). It remained predefined levels before GTx (P>0.05), and was not significantly increased after NavX-guided gene therapy (P>0.05, Table 2). During mapping and injection, no animal had pericardial effusion or malignant arrhythmia (Fig. 2C, 2D). No animal died as a result of the mapping and injection procedures. During mapping and injection, the doses and time of X-ray radiation were recorded. The average time was 14.54±4.47 min, and the average doses of radiation were 275.17±162.16 mGy.

**Table 2:** The analyses of clinical chemistry.

G	T	WBC(1×10^9^/L)	ALT(U/L)	AST(U/L)	BUN(mmol/L)	Cr(µmol/L)	CK-MB(U/L)
S	B	8.36±0.99	29.60±5.13	27.80±3.70	6.44±0.99	95.60±8.85	23.60±1.68
	L1 h	8.02±0.60	35.20±8.32	31.20±5.97	6.00±1.29	95.00±9.75	41.80±8.81
	L6 h	7.38±1.61	31.00±10.02	27.80±4.76	6.00±0.65	88.00±11.64	81.60±18.06*
	L24 h	7.80±1,10	32.20±4.09	33.40±6.02	6.24±0.99	88.00±3.61	200.60±28.85*
	L3dT0 h	8.50±0.467.50±1.59	32.80±8.6734.00±7.29	27.20±4.0934.83±4.45	5.52±0.895.95±0.67	86.40±7.0287.67±17.37	82.20±21.22*25.67±3.78
	T24 h	8.66±0.82	33.00±4.06	31.60±5.13	5.86±0.77	85.40±6.77	25.40±8.71
	T3 d	7.26±1.77	27.00±10.51	27.60±4.45	6.32±0.61	85.00±8.25	24.00±9.56
	T4 w	7.26±1.01	30.40±5.98	34.00±5.66	5.96±1.03	85.80±9.36	26.40±3.05
H	B	7.89±2.07	33.16±7.68	34.50±7.15	5.85±0.96	88.67±17.59	24.83±3.87
	L1 h	7.72±2.00	30.00±6.63	32.50±6.78	6.00±1.05	89.00±15.72	36.33±4.27
	L6 h	6.43±1.28	38.00±7.89	33.50±4.58	6.75±0.99	94.17±8.04	85.50±13.14*
	L24 h	7.30±0.99	30.83±7.73	33.50±4.55	5.80±0.48	80.33±12.34	173.17±27.25*
	L3 dT0 h	7.45±1.608.28±0.63	29.50±8.1731.00±5.81	34.50±6.9532.40±3.87	6.68±0.696.24±4.80	85.17±12.8395.60±8.85	93.83±17.81*24.60±5.32
	T24 h	7.52±1.32	32.00±6.42	33.00±6.07	5.70±0.91	83.67±10.73	26.17±4.88
	T3 d	7.85±0.65	31.50±7.39	31.33±6.59	6.05±0.94	90.67±10.80	25.17±4.62
	T4 w	6.97±1.25	33.50±6.22	33.83±4.31	6.30±1.12	91.00±9.08	24.83±5.53

G: groups; S: saline; H: HGF; T: time; B: before the LAD ligation; L1 h, L6 H, L24 h, L3 d: 1, 6, 24 hours and3 days after the LAD ligation; T0 h: before the injection; T24 h, T3 d, T4 w: 24 hours, 3 days and 4 weeks after the injection. *P<0.01 vs. before the ligation.

### Reduction of Infarct Size and Increase of Myocardial Voltage

**Figure 3:**
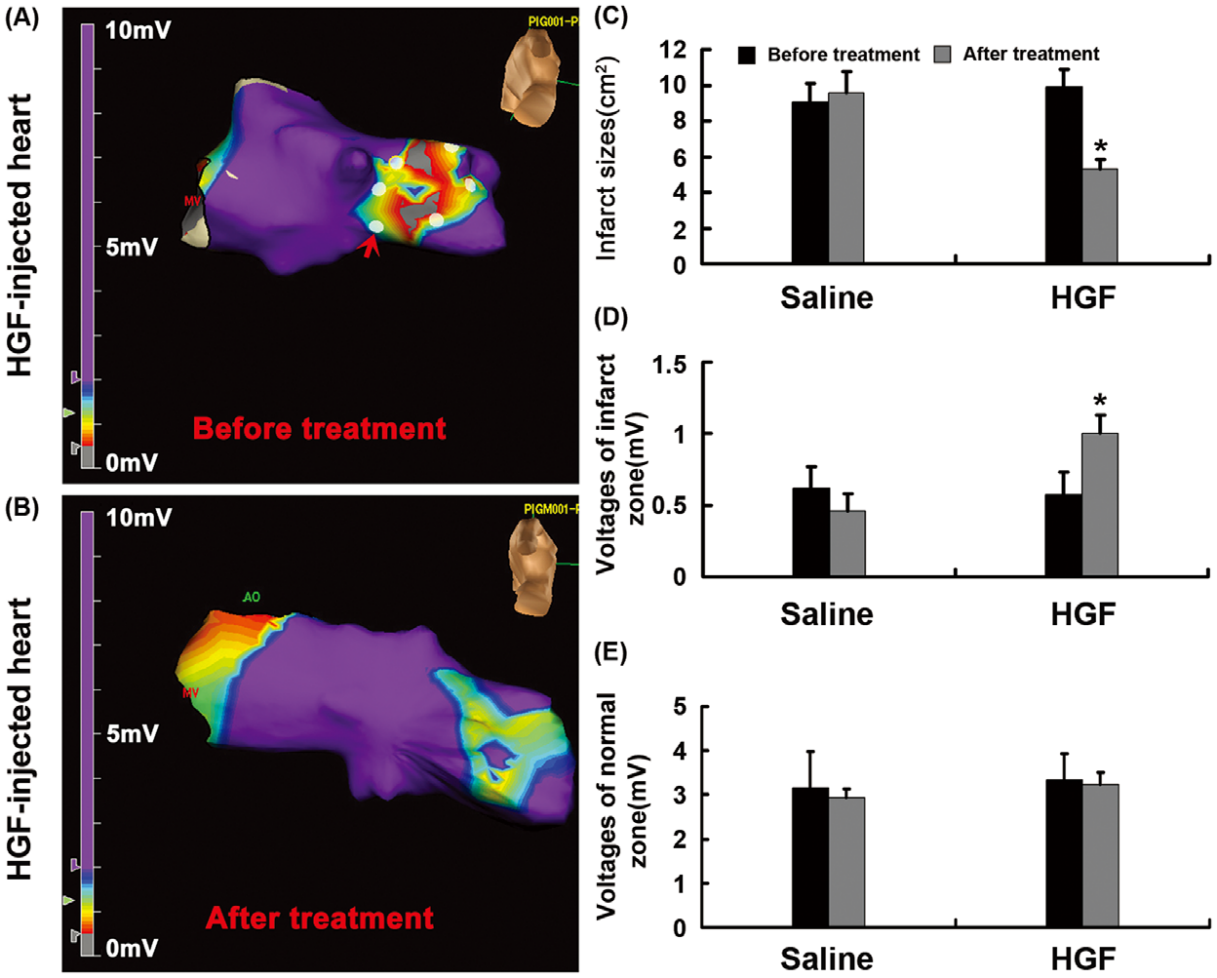
Infarct size and myocardial voltage. (A), (B) the left ventricular geometry before and after the GTx. Gray and purple respectively indicates the myocardial infarct and normal zones; the region between gray and purple is transitional zone. Red arrow indicates injected site marked with white dots. (C) Bar graph showed the infarct sizes significantly reduced in the HGF-injected hearts after GTx than those before GTx. (D), (E) Bar graph explained the myocardial voltage levels in the infarct and normal zones of HGF-injected hearts. There were significantly higher myocardial voltage levels in the infarct zone of HGF-injected hearts after GTx than those before GTx. *P<0.01 vs. before GTx in HGF group.

Local electrograms were recorded with NavX before and 4 weeks after GTx. There were significantly higher myocardial voltage levels in infarct zones of HGF-injected hearts after GTx (1.00±0.13 mV) than that before GTx (0.51±0.16 mV) (Fig. 3A, 3D, P<0.01). However, the myocardial voltage level of infarct zones have no significant difference before and after injection (0.62±0.1 versus 0.46±0.12 mV, P>0.05) in saline groups. In the zones of normal myocardium, voltage levels did not significantly change before and after the injection between two groups (Fig. 2A, 2E, P>0.05). Accordingly, infarct sizes significantly reduced in the HGF-injected hearts after GTx (5.28±0.55 cm^2^) than those before GTx (9.93±0.98 cm^2^) (P<0.01, std error: 0.39 and 0.26, 95% confidence intervals).But no significant difference was found before and after saline injection (9.06±1.06 versus 9.58±1.15 cm^2^) in the hearts of control group (Fig. 3A, 3B, P = 0.48, std error: 0.47 and 0.52, 95% confidence intervals). The infarct sizes significantly reduced in the HGF-injected hearts after GTx (5.28±0.55 cm^2^) compared to those (9.06±1.06 cm^2^) in the hearts of control group.

### Improvement of Myocardial Perfusion and Cardiac Function

**Figure 4:**
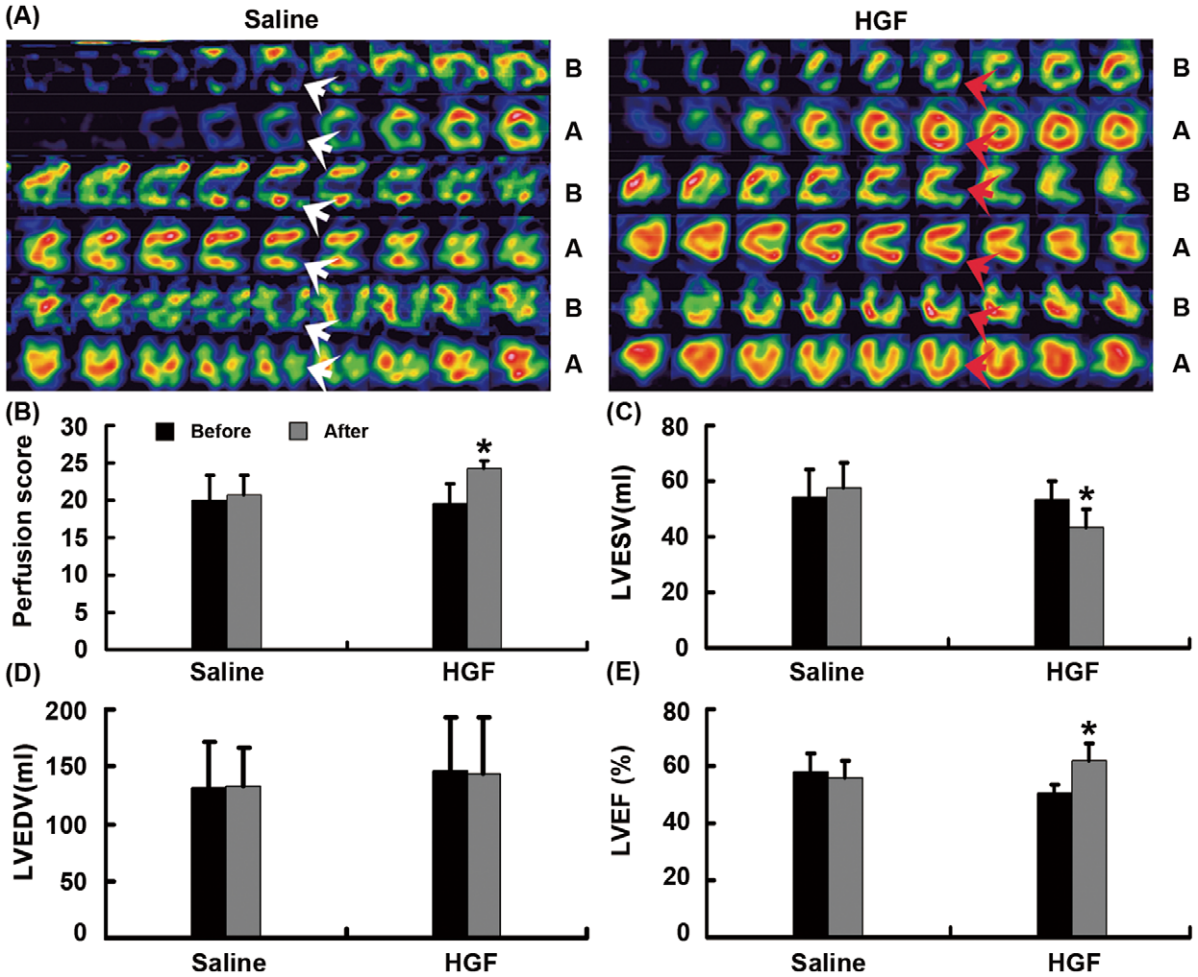
The myocardial perfusion and cardiac function. (A) Representative images of SPECT of saline (left) and HGF (right) treated porcine chronic myocardial infarct heart. Red arrows indicate regions with better myocardial perfusion; white arrows indicate regions that had similar myocardial perfusion. (B) Bar graph showing myocardial perfusion score, *P = 0.001 vs. four weeks after vector injection; (C) LVESV, *P<0.01 vs. four weeks after vector injection; (D) LVEDV, there was significant difference four weeks after the injection compared to that before the injection, P>0.05. (E) LVEF, *P<0.01 vs. four weeks after vector injection; B: before GTx; A: four weeks after GTx.

SPECT was used to detect myocardial perfusion scores and cardiac function. The myocardial perfusion scores in the HGF group were significantly improved 4 weeks after GTx compared to those before GTx (P = 0.001, Fig. 4A, 4B). The perfusion scores of control group have no significant difference before and after saline injection (P>0.05, Fig. 4A, 4B). The HGF group had greater LVEF and lower LVESV four weeks after GTx compared to that before GTx (P<0.05, Fig. 4A, 4C, 4D).

### HGF Expression

**Figure 5:**
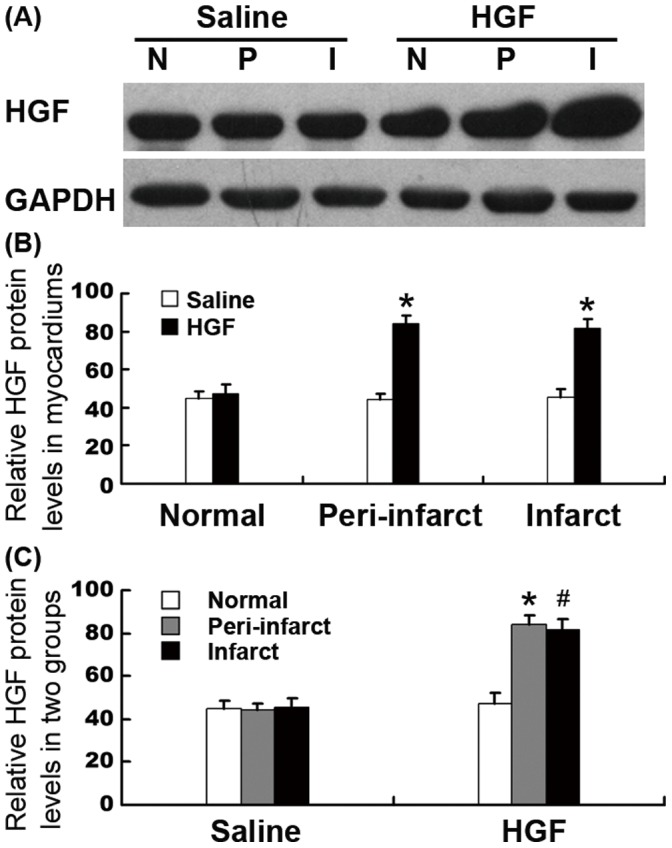
HGF expression. (A) Representative image of HGF expression. (B) Bar graph showed HGF protein had higher expression in the infarct and peri-infarct zone of the treatment group compared to that in saline group (*P<0.01). (C) In HGF group, the myocardial infarct and peri-infarct zone had higher HGF expression than the normal zone (*P<0.01, ^#^P<0.01). I, infarct zone; P, peri-infarct zone; N, normal zone.

To investigate whether the adenovirus could mediate HGF expression *in vivo*, Western blots were performed 4 weeks after GTx. There was higher HGF expression in the myocardial infarct and peri-infarct zones of treatment group than that in control group (Fig. 5A, 5C, P<0.01). In the HGF group, the myocardial infarct and peri-infarct zone had higher HGF expression compared with the normal zone (P<0.01), and the normal zone was lowest in each of the three myocardial zones (Fig. 5A, 5B, P<0.01). These results indicated that the HGF gene expression could be mediated by the adenovirus vector via our injection system guided by NavX in this porcine chronic myocardial ischemia model.

### Increase of Vascular Density in Myocardial Infarct and Peri-infarct Zones

**Figure 6:**
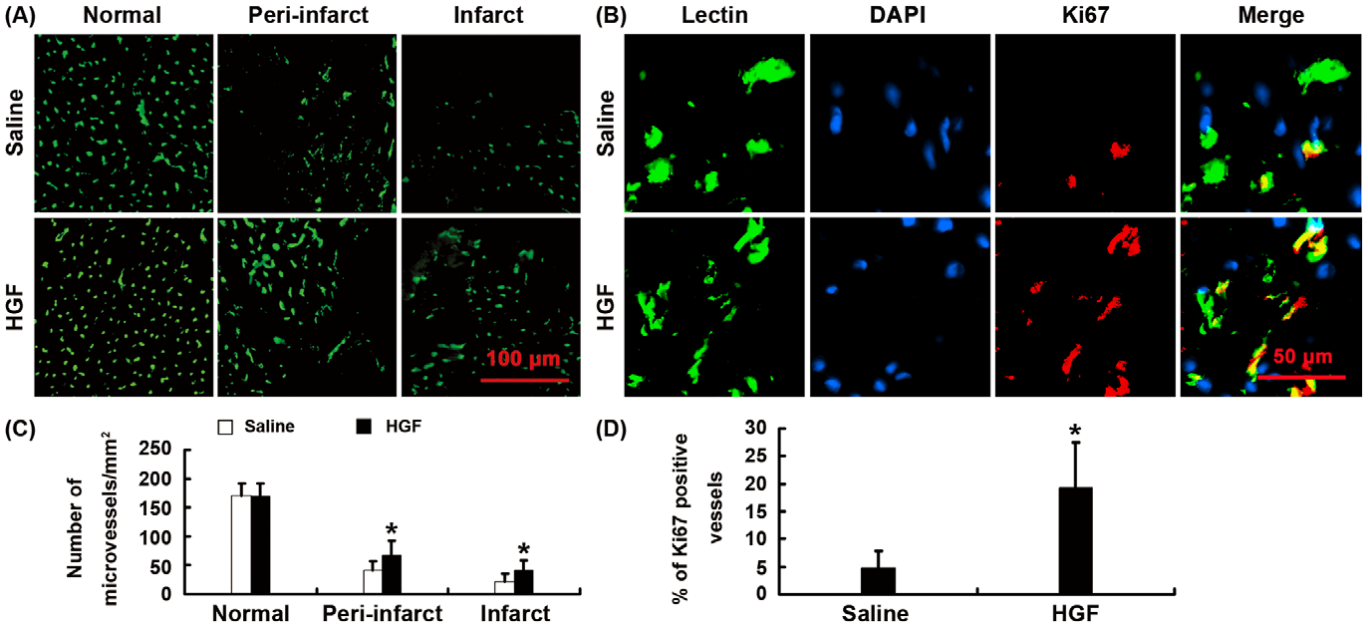
The vascular density. (A) Representative pictures of Lectin stained sections. (B) The immunofluorescence of the Ki67-positive cells in the border zone of the infarct. (C) Bar graph showing the quantification of microvessels. (D) Bar graph showing the quantification of Ki67-positive vessels. *P<0.01 vs. saline group.

To analyze the angiogenesis mediated by expression of HGF, microvessels were counted blindly on lectin stained sections. The HGF group had significantly more capillaries in the myocardial infarct (41.10±17.52/mm^2^) and peri-infarct (68.20±23.14/mm^2^) zones than that in control group (22.20±12.56, 41.75±15.11/mm^2^) (Fig. 6A, 6C, P<0.01).

To identify newly formed blood vessels, we used an antibody specific to Ki67 to examine proliferating endothelial cells. We detected significantly more Ki67 positive endothelial cells in the HGF-injected hearts (19.25±8.17/mm^2^) than that in the saline-injected hearts (4.70±3.08/mm^2^) (Fig. 6B, 6D, P<0.01). Thus, over-expression of HGF induced active angiogenesis.

## Discussion

Vector-mediated cardiac gene therapy holds tremendous promise as a translatable platform technology for treating many cardiovascular diseases, but its application has been hindered due to the lack of an acknowledged ideal delivery method in a large animal model. We report here a safe, effective, practical and relatively non-invasive protocol in the pig which results in therapeutic gene transfer mediated by adenovirus following delivery guided by NavX system. This procedure is superior to previously reported large animal cardiac gene transfer techniques because it combines the advantages of direct intramyocardial injection with those of a non-invasive percutaneous delivery system.

The phase I clinical study showed that it is safe and effective to use an adenoviral gene-transfer vector to deliver the human HGF to individuals with clinically significant coronary artery disease [7]. Although the use of a mini-thoracotomy seems to be generally well tolerated, even in patients with advanced myocardial ischemia, nevertheless, the procedure has some risk associated with the administration of general anesthesia and some morbidity associated with surgical manipulation (particularly in patients with previous bypass surgery), and it limits the feasibility of repeat administrations [17]. Our previous animal studies have proven the efficiency of activating angiogenesis and improving cardiac function by direct intramyocardial delivery of HGF into the myocardium following open chest thoracotomy [18]. However, one pig died at week 3 after the second operation, while all the pigs in the present study survived till the end of the observation period.

Currently, there are several ongoing preclinical studies and clinical trails evaluating the therapeutic efficacy of cardiac gene transfer as a novel treatment for failure heart or ischemic heart disease. The feasibility of in vivo cardiac gene transfer with the aid of percutaneous catheter-mediated intracoronary delivery has been demonstrated in many studies, and some authors believe that it is the most clinically relevant method because of the possibility of delivering vectors to the whole myocardium and because of the extensive clinical experience in coronary catheterization procedures [19], [20], [21]. However, the efficiency of adenovirus and adeno-associated virus-mediated gene delivery by this method is highly variable among studies [22]. In addition, coronary perfusion would have limited utility in ischemic heart disease, because blood flow would be limited in the targeted areas of the heart. Recently, the catheter-based injection of fluoroscopic guidance was reported in several studies [23], [24]. Although this method is relatively non-invasive, it requires contrast media added to the vector solution so that injection sites could be visualized. In addition, the injections need a long time exposed to X-ray. Radiation can cause a small increase in the risk of fatal malignancy for both the patient and electrophysiologist [25]. The intramyocardial injection strategy has been substantially improved by the introduction of the NOGA electromechanical mapping [26], [27], which offers a endocardial route to myocardial transduction as efficient as injections via thoracotomy [28]. The transendocardial injections guided by NOGA left ventricular electromechanical mapping provided an efficient, safe and practical technique, however average mapping(15±6 min) and injection time (23±6 min) guided by NOGA [29]was longer than the total time of mapping and injection (14.54±4.47 min) in our study. The reason is that two catheters were using during mapping and injection guided by NOGA system. After the procedure of mapping, the injection may be executed with the injection catheter replaced mapping catheter. Interestingly, the catheter in our study has the two characteristics of injection and signal detection, because the distal tip of injection catheter is fixed with a headend and a three-loop electrode to detect local signals. When mapping was completed, the injection may be processed at once without changing catheter. So the time of operation may be reduced compared with that based NOGA system, and the operation risk would be decreased. In summary, we believe that both coronary perfusion and endocardial gene transfer are delivery methods with potential clinical utility and that future investigation should be directed at comparing these methods in large animal model.

Moreover, our delivery method is safe and relatively non-invasive, suggesting it would be better tolerated in patients with significant heart disease than procedures requiring thoracotomy, cardiopulmonary bypass. In this study, all animals survived the procedures of mapping and injection. WBC, ALT, AST, Bun and Cr levels were not significantly changed 24 hours, 3 days and 4 weeks after vector injection compared to those before the LAD ligation. The increase of CK-MB level showed that the LAD ligation led to ischemic myocardial injury 6, 24 hours, and 3 days after the ligation. However, there was no significant increase in CK-MB 24 hours, 3 days and 4 weeks after the GTx compared to baseline values. During mapping and injection, no animal had pericardial effusion or malignant arrhythmia. These results indicated that the method of the GTx did not lead to the detectable injury of kidney, liver,myocardium, pericardial effusion or malignant arrhythmia.

Furthermore, the effective delivery guided by NavX system was identified in the present study. The myocardial infarct and peri-infarct zones had higher HGF expression compared with the normal zone, and the normal zone was the lowest among the three myocardial zones in the HGF group. The cardiac function, myocardial perfusion scores and voltage of the HGF group improved significantly, and infarction size reduced significantly four weeks after the GTx compared to those before the GTx. Our previous studies have shown that over-expression of HGF not only could induce active angiogenesis, but reduce cardiomyocyte apoptosis and proliferation in the myocardial infarct and peri-infarct zones [30]. Taniyamn Y et.al have reported that increase in local HGF expression may participate in the prevention of myocardial injury by Ang II blockade through its antifibrotic action [31]. Thus, angiogenesis, apoptosis reduction, proliferation and antifibrotic activities with the endocardial HGF transfer could lead to improvement of cardiac function in HGF-injected heart.

The limitations of this study: To investigate the efficiency and safety of the intramyocardial injection guided by NavX in the study, we need a group that should be injected with Ad-HGF via an intracoronary arterial route or surgical epicardial approach individually. However, we did not have such a group in this experiment. Thus the effect of the HGF transfer will be compared to individual transfer in the future. CK-MB is not a perfect marker of myocardial injury, and other more specific and sensitive markers such as troponin should be used for future studies. The optimal mapping technique, while preserving accuracy [32], achieves high density with short acquisition times. The number of points mapped by NavX was relatively limited due to the unavailability of Duo-Decapolar catheter. Although Ad–based gene therapy is a promising technique for the delivery of growth factors, Ads could activate the innate immune system [33], [34]. In the present study, local inflammation caused by Ads was not detected. An understanding of the innate response to Ads is important to overcome the last remaining hurdle to improve the safety and effectiveness of these agents, the immune response should be focus in future study.

To sum up, adenoviral gene transfer of HGF used in our catheter-based intramyocardial injection guided by NavX is a feasible and safe approach for the induction of angiogenesis, the increase of myocardial voltage and the reduction of infarct size that could lead to the improvement of cardiac function and myocardial perfusion. Thus, this approach may provide a safe and effective angiogenic gene therapy system for ischemic heart disease in potential clinical application.
